# Critical evaluation of factorial experimental designs and temporal confounding in comparative analysis of dietary vs parenteral iron administration effects on cortical and trabecular bone parameters

**DOI:** 10.1093/jbmrpl/ziaf184

**Published:** 2025-12-04

**Authors:** Muhammad Hammad Zaheer, Zainab Shahnawaz, Hamza Zaheer

**Affiliations:** Allama Iqbal Medical College, Lahore, Punjab 54700, Pakistan; Allama Iqbal Medical College, Lahore, Punjab 54700, Pakistan; Services Institute of Medical Sciences, Lahore, Punjab 54000, Pakistan

**Keywords:** Iron overload, bone health, cortical bone loss, trabecular bone parameters, factorial design, statistical methodology, temporal confounding, multiple comparisons, parenteral iron, dietary iron

Dear Editor,

We read with great interest the article by Steele-Perkins et al. entitled “Comparison between dietary, parenteral, and genetic iron overload on bone health reveals secondary iron overload as a driver of cortical bone loss and fracture risk in mice”*.*^1^ The study offers valuable insights into the skeletal consequences of different modes of iron overload. However, we would like to highlight several methodological and statistical issues that may limit the robustness of the conclusions.

The study’s statistical approach raises significant concerns regarding the reliability of findings. With experimental groups containing only 7-10 animals per condition, the authors provide no power calculations to justify adequate sample sizes for detecting meaningful differences.^2^ More problematically, numerous bone parameters were analyzed using simple *t*-tests and Mann–Whitney tests across multiple groups without correction for multiple comparisons. This approach substantially inflates the risk of false positives, particularly given the extensive testing of trabecular parameters, cortical measurements, mechanical properties, and blood indices. The absence of Bonferroni, Holm-Šidák, or false discovery rate corrections undermines confidence in the reported significant differences.^3^ Additionally, the use of parametric tests with such small sample sizes may be inappropriate, as groups with as few as 7 animals have insufficient power for reliable normality testing, yet the authors applied unpaired *t*-tests based on Shapiro–Wilk results. Nonparametric approaches or mixed-model analyses would provide more robust statistical inference with these sample sizes.

The experimental design fails to capitalize on its factorial structure. Rather than employing appropriate factorial ANOVA or mixed-model approaches to examine the stated research question about genotype–intervention interactions, the authors relied exclusively on pairwise comparisons.^4^ This analytical choice obscures potential interaction effects between genetic background and iron overload modality, which appears central to understanding differential bone responses across conditions.

The study’s temporal design introduces confounding variables that complicate interpretation. Dietary iron overload was administered for 30 wk, while parenteral iron treatment lasted only 8 wk, yet the authors directly compare outcomes between these interventions. This duration mismatch makes it impossible to distinguish whether observed differences reflect treatment modality or exposure time, fundamentally limiting the study’s comparative value.

Additionally, the use of parametric tests with small sample sizes may be inappropriate. Groups with as few as 7 animals have insufficient power for reliable normality testing, yet the authors applied unpaired *t*-tests based on Shapiro–Wilk results. Nonparametric approaches or mixed-model analyses would provide more robust statistical inference with these sample sizes.

Finally, while the authors acknowledge using the ROUT method for outlier removal, they do not report the number of excluded animals or whether exclusions disproportionately affected specific experimental groups, potentially introducing selection bias.

These methodological limitations suggest that the conclusions regarding differential bone effects of iron overload modalities should be interpreted cautiously. Future studies would benefit from adequate power calculations, appropriate correction for multiple testing, factorial statistical designs matching the experimental structure, and standardized treatment durations to enable meaningful comparisons.
